# Novel GLA variant (c.752A>C; p.Glu251Ala) identified in a patient with Fabry cardiomyopathy and familial segregation

**DOI:** 10.3389/fmolb.2026.1828669

**Published:** 2026-08-20

**Authors:** Ting Zhang, Sulan Huang, Fan Huang

**Affiliations:** 1 Department of Cardiology, Changde Hospital, Xiangya School of Medicine, Central South University (The First People’s Hospital of Changde City), Changde, China; 2 Department of Radiology, Changde Hospital, Xiangya School of Medicine, Central South University (The First People’s Hospital of Changde City), Changde, China

**Keywords:** Fabry disease (FD), gene mutation, GLA, globotriaosylsphingosine (lyso-Gb3), hypertrophic cardiomyopathy, α-galactosidase(α-Gal A)

## Abstract

**Background:**

Fabry disease (Anderson–Fabry disease,FD) is an X-linked lysosomal storage disorder caused by variants in GLA, resulting in α-galactosidase A (α-Gal A) deficiency and accumulation of globotriaosylsphingosine (lyso-Gb3), with frequent cardiac involvement.

**Case Summary:**

A 53-year-old man presented with exertional chest tightness and dyspnea. Electrocardiography and echocardiography demonstrated marked left ventricular hypertrophy. Cardiac magnetic resonance revealed diffuse hypertrophy with extensive subendocardial late gadolinium enhancement. ^99mTc-pyrophosphate scintigraphy showed minimal myocardial uptake, arguing against transthyretin cardiac amyloidosis. Biochemical testing showed markedly reduced α-Gal A activity and elevated lyso-Gb3 levels. Whole-exome sequencing identified a previously unreported GLA variant (NM_000169.3:c.752A>C; p.Glu251Ala), which was confirmed by Sanger sequencing and detected in multiple family members. The variant is currently classified as a variant of uncertain significance (VUS). Familial analysis demonstrated a segregation pattern consistent with X-linked inheritance.

**Conclusion:**

We report a novel GLA variant associated with biochemical abnormalities and familial segregation consistent with FD. These findings support a potential role of this variant in Fabry cardiomyopathy and expand the mutational spectrum of GLA, although its pathogenicity requires further validation.

## Introduction

Fabry disease (FD), also known as Anderson–Fabry disease, is an X-linked lysosomal storage disorder caused by variants in the GLA gene located on chromosome Xq22.1. These variants result in partial or complete deficiency of α-galactosidase A (α-Gal A), leading to the progressive accumulation of its metabolic substrates, globotriaosylceramide (Gb3) and its derivative globotriaosylsphingosine (lyso-Gb3), in multiple tissues including the nervous system, skin, kidneys, and heart. This progressive accumulation leads to multisystem organ damage and a wide spectrum of clinical manifestations (Germain, 2010). In the cardiovascular system, the accumulation of Gb3 and lyso-Gb3 in cardiomyocytes can lead to left ventricular hypertrophy (LVH), conduction abnormalities, arrhythmias, and dysfunction of the aortic and mitral valves (Pieroni et al., 2021). Cardiac involvement is a major determinant of prognosis in FD and represents one of the leading causes of morbidity and mortality in adult patients (Desnick et al., 2003). FD is generally classified into classic and late-onset forms. The classic phenotype is more common in males and is characterized by severely reduced α-Gal A activity (<1% of normal), typically with onset in childhood or adolescence. Clinical manifestations include peripheral neuropathy (e.g., limb pain), hypohidrosis or anhidrosis, angiokeratomas, and gastrointestinal symptoms. With disease progression, patients may develop renal dysfunction, cardiac involvement (LVH, conduction disorders, heart failure), and cerebrovascular complications such as transient ischemic attack or stroke. Late-onset FD is more commonly recognized in adults and may present with residual enzyme activity, often predominantly affecting the heart and/or kidneys (Kok et al., 2021; Azevedo et al., 2021). Notably, late-onset cardiac variants are increasingly identified among patients with unexplained LVH, with a reported prevalence of approximately 0.5%–1% in selected LVH cohorts (Nakao et al., 1995). Given the nonspecific nature of cardiac manifestations, FD is often underdiagnosed or misdiagnosed, particularly in patients presenting with isolated LVH. Recent consensus recommendations emphasize the importance of systematic screening for FD in patients with unexplained LVH, incorporating multimodal approaches including cardiac imaging, enzymatic assays, biomarker assessment (e.g., lyso-Gb3), and genetic testing (Nordin et al., 2019a; Schiffmann, 2009). Early diagnosis is critical, as disease-specific therapies such as enzyme replacement therapy (ERT) or chaperone therapy can delay disease progression and improve clinical outcomes. Among these approaches, GLA gene analysis plays a crucial role in the diagnosis of FD and in family screening, particularly in female patients in whom α-Gal A activity may be normal due to X-chromosome inactivation (Amodio et al., 2022). In addition, identification of disease-associated variants enables cascade screening of family members.

This case report describes a patient with unexplained LVH in whom genetic testing identified a previously unreported GLA variant. Familial investigation further supported its segregation within the family. This variant has not been previously reported in available databases or literature and may represent a rare genetic variant potentially associated with FD, although further functional and clinical evidence is required to confirm its pathogenicity.

## Methods (case summary and diagnostic workup)

A 53-year-old man was admitted to the Department of Cardiology, The First People’s Hospital of Changde City, with a 2-month history of chest tightness and shortness of breath that worsened with exertion and resolved within a few minutes of rest. He also reported fatigue and palpitations but denied syncope, fever, cough, nausea, or vomiting. He had a history of schistosomiasis but no known history of hypertension, coronary artery disease, diabetes mellitus, chronic hepatitis B, or tuberculosis. He was married and had two daughters; both parents were deceased from unknown causes. One of his two older brothers had a history of hypertrophic cardiomyopathy.

On physical examination, his heart rate was 53 beats/min, blood pressure was 132/82 mmHg, and body mass index (BMI) was 18.8 kg/m^2^. No cardiac murmur was detected. Laboratory testing indicated preserved renal function (serum creatinine: 73 μmol/L; reference range: 57–97 μmol/L). However, cardiac biomarkers were elevated, including cardiac troponin I (cTnI: 0.191 ng/mL; reference range: 0.000–0.026 ng/mL) and N-terminal pro–B-type natriuretic peptide (NT-proBNP: 2817 pg/mL; reference range: 0–900 pg/mL). Serum immunofixation electrophoresis and qualitative urine testing for Bence Jones protein were negative.

**FIGURE 1:**
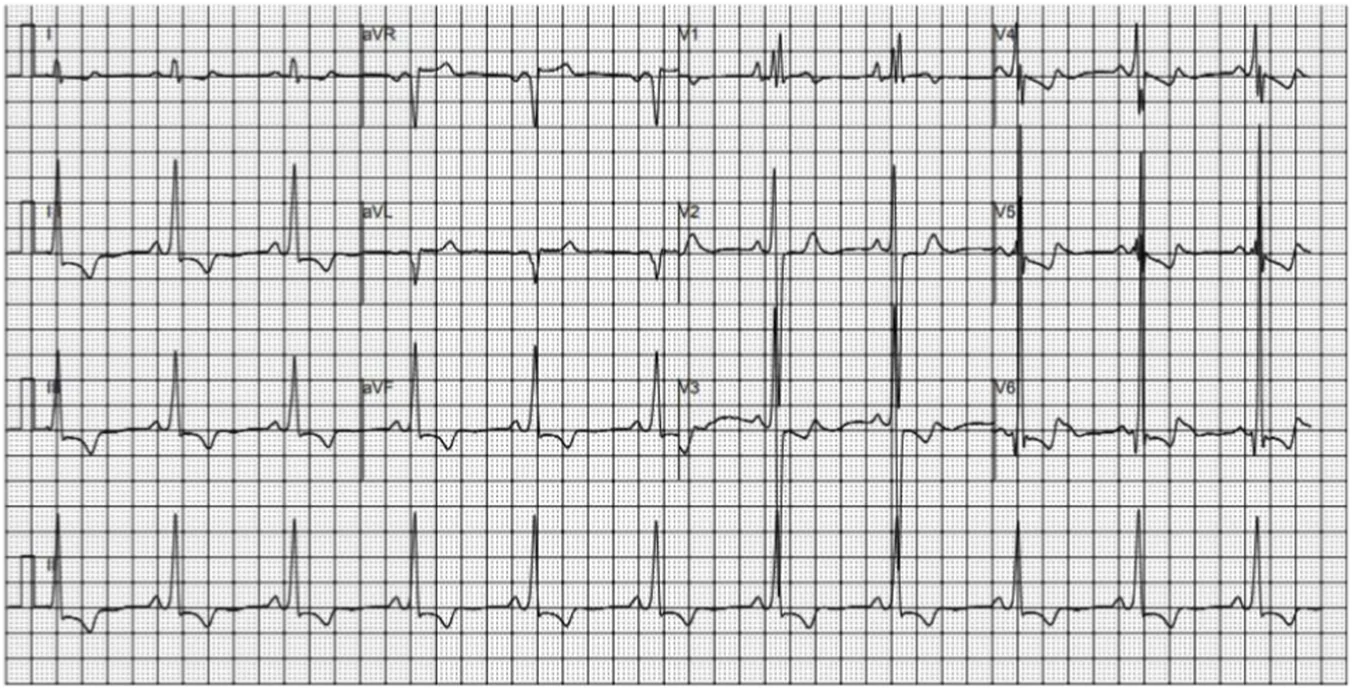
Electrocardiogram (ECG) on admission. Notes: The ECG shows sinus bradycardia, complete right bundle branch block, abnormal positive IPIV1, and left ventricular enlargement with ST-T changes.

**FIGURE 2:**
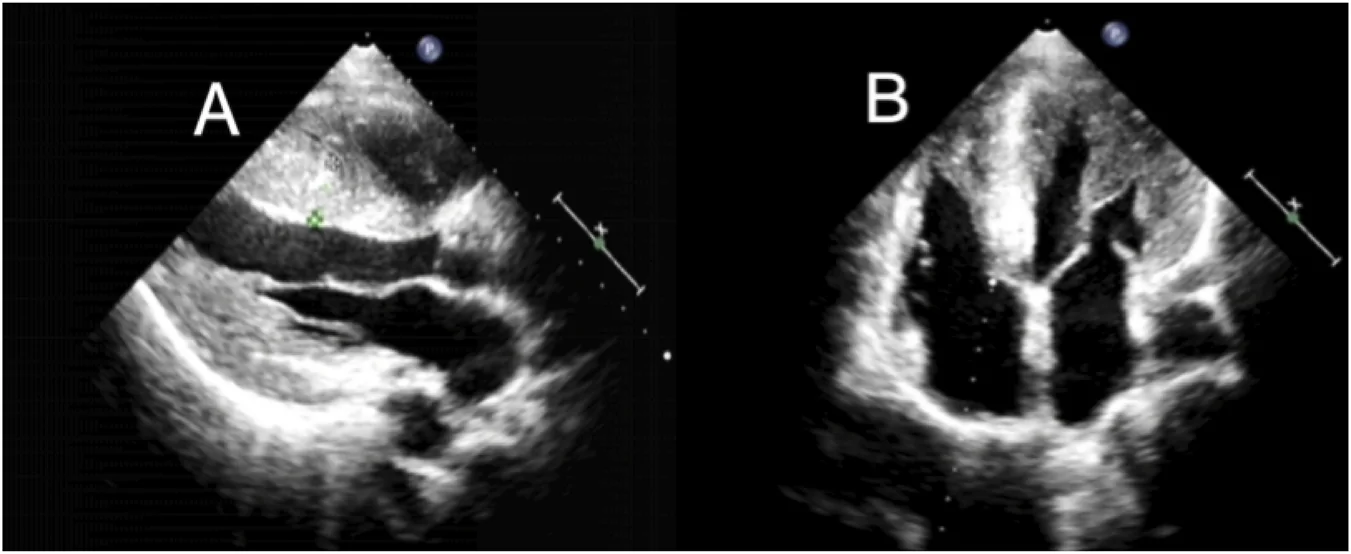
Transthoracic echocardiography (TTE) of the proband. Notes: Echocardiography reveals significant and asymmetric left ventricular hypertrophy, as demonstrated in the left ventricular short-axis **(A)** and four-chamber views **(B)**. The maximal wall thickness is 18 mm at the interventricular septum and 18 mm at the posterior wall.

**FIGURE 3:**
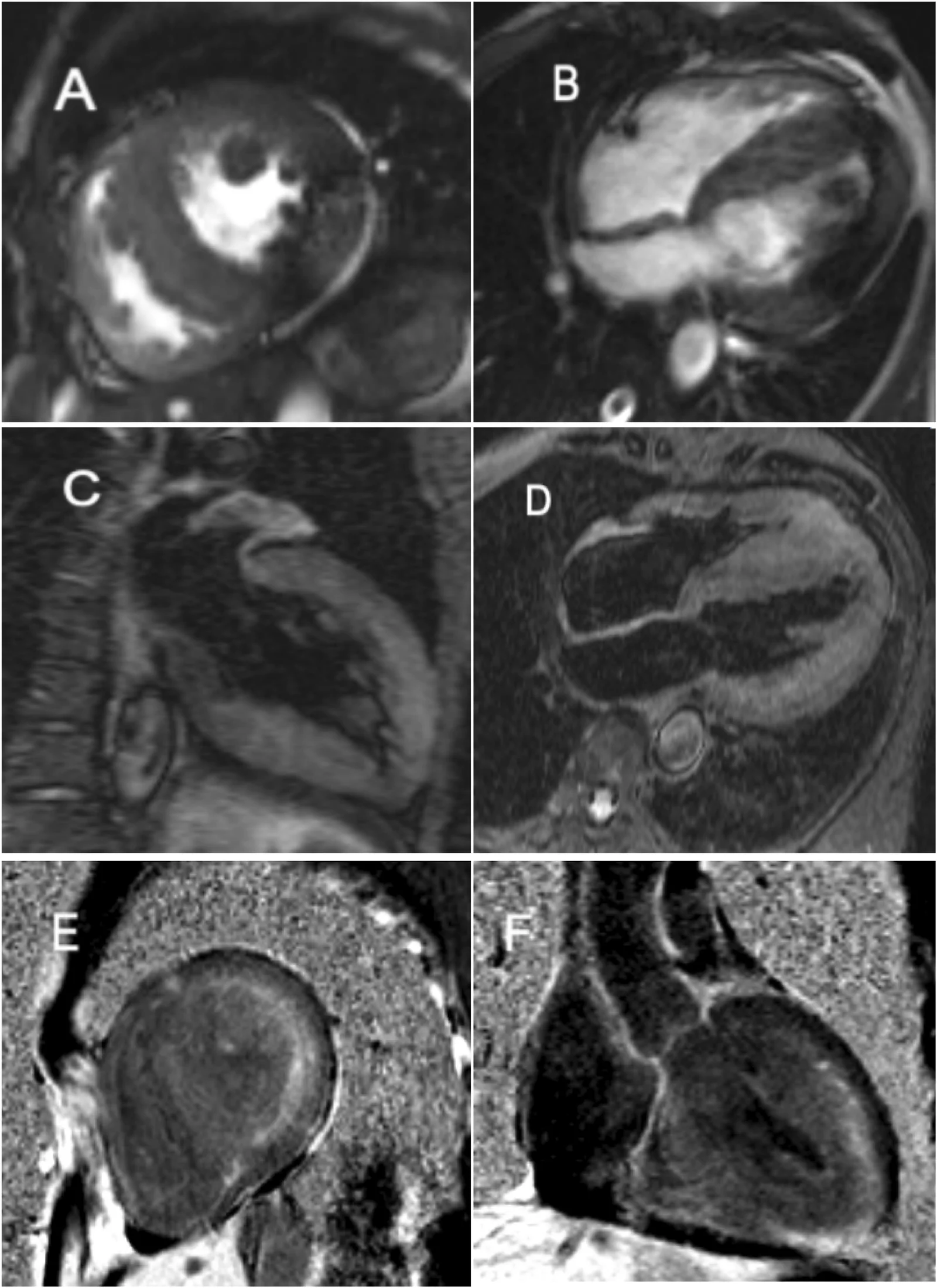
Cardiac magnetic resonance imaging (CMR) of the proband. Notes: Cardiac MRI (3.0 T) cine images (balanced steady-state free precession sequence) in the basal left ventricular short-axis **(A)** and four-chamber views **(B)** demonstrate marked asymmetric left ventricular hypertrophy (maximal wall thickness: 21 mm in the septum and 19 mm in the lateral wall). T2-weighted fat-suppressed images in the basal two-chamber **(C)** and four-chamber views **(D)** show myocardial edema. Late gadolinium enhancement (LGE) images in the basal short-axis **(E)** and four-chamber views **(F)** demonstrate extensive subendocardial fibrosis of the left ventricular wall, with a zebra sign in the interventricular septum.

The electrocardiogram (ECG) (Figure 1) showed sinus bradycardia, complete right bundle branch block, increased positive terminal force in lead V1 (PTF-V1), and electrocardiographic evidence of LVH with ST–T abnormalities. Transthoracic echocardiography (Figure 2) revealed marked left ventricular wall thickening, with interventricular septal thickness of 17 mm (basal), 18 mm (mid), and 18 mm (apical), and a posterior wall thickness of 18 mm. Mild mitral regurgitation and mild tricuspid regurgitation were present, and the left ventricular ejection fraction was 75%. Cardiac magnetic resonance (CMR) imaging (Figure 3) demonstrated diffuse LVH with extensive subendocardial late gadolinium enhancement (LGE). The interventricular septum measured approximately 20–21 mm, and the left ventricular lateral wall measured 18–19 mm. LGE was also observed in the right ventricular wall, interatrial septum, and atrioventricular valves.

These imaging findings were suggestive of cardiac amyloidosis; however, other genetic and metabolic cardiomyopathies remained in the differential diagnosis. To further evaluate for amyloidosis, 99mTc-pyrophosphate (PYP) scintigraphy was performed and showed minimal myocardial uptake (visual grade 1), which did not meet diagnostic criteria for transthyretin (ATTR) cardiac amyloidosis.

To further investigate a potential inherited metabolic cardiomyopathy, enzymatic and genetic analyses were performed. Plasma α-Gal A activity was measured in peripheral blood samples using a validated colorimetric assay based on synthetic substrate hydrolysis, according to the manufacturer’s protocol. Enzyme activity was quantified by measuring the rate of substrate conversion and expressed as nmol/h/mg protein, with interpretation based on support ed reference ranges. In addition, plasma lyso-Gb3 levels were quantified as a disease-specific biomarker using high-performance liquid chromatography coupled with tandem mass spectrometry (LC–MS/MS). Plasma samples were collected using heparin anticoagulation and processed according to standard laboratory protocols. Results were interpreted according to laboratory-specific reference intervals.

For the proband and most family members, enzymatic assays, lyso-Gb3 measurement, and genetic testing were performed at KingMed Diagnostics (Guangzhou, China), an accredited clinical laboratory. In one additional family member, enzymatic, biomarker, and genetic analyses were independently conducted at Revvity Medical Laboratory (Suzhou, China). Genomic DNA was extracted from peripheral blood samples. Targeted next-generation sequencing (NGS) of the GLA gene was performed, covering coding exons and flanking intronic regions. Sequencing data were aligned to the human reference genome (GRCh37/hg19), followed by variant calling and annotation using standard bioinformatics pipelines. Identified variants were filtered and evaluated against multiple population and disease databases, including gnomAD, the 1000 Genomes Project, the Exome Sequencing Project (ESP), and ClinVar. In addition, available literature and database evidence were reviewed to support variant interpretation. Variants were classified according to the American College of Medical Genetics and Genomics/Association for Molecular Pathology (ACMG/AMP) guidelines. The candidate variant was validated by Sanger sequencing. Targeted Sanger sequencing was subsequently performed in available family members to assess co-segregation of the variant with the clinical phenotype. Sanger sequencing primarily covered exonic regions and exon–intron boundaries and is suitable for detecting single nucleotide variants and small insertions/deletions, but may have limitations in detecting large structural variants. All analyses were performed in accordance with standard laboratory quality control procedures. Copy number variation (CNV) analysis was not performed as part of the genetic evaluation in this study.

## Results

### Genetic findings

**FIGURE 4:**
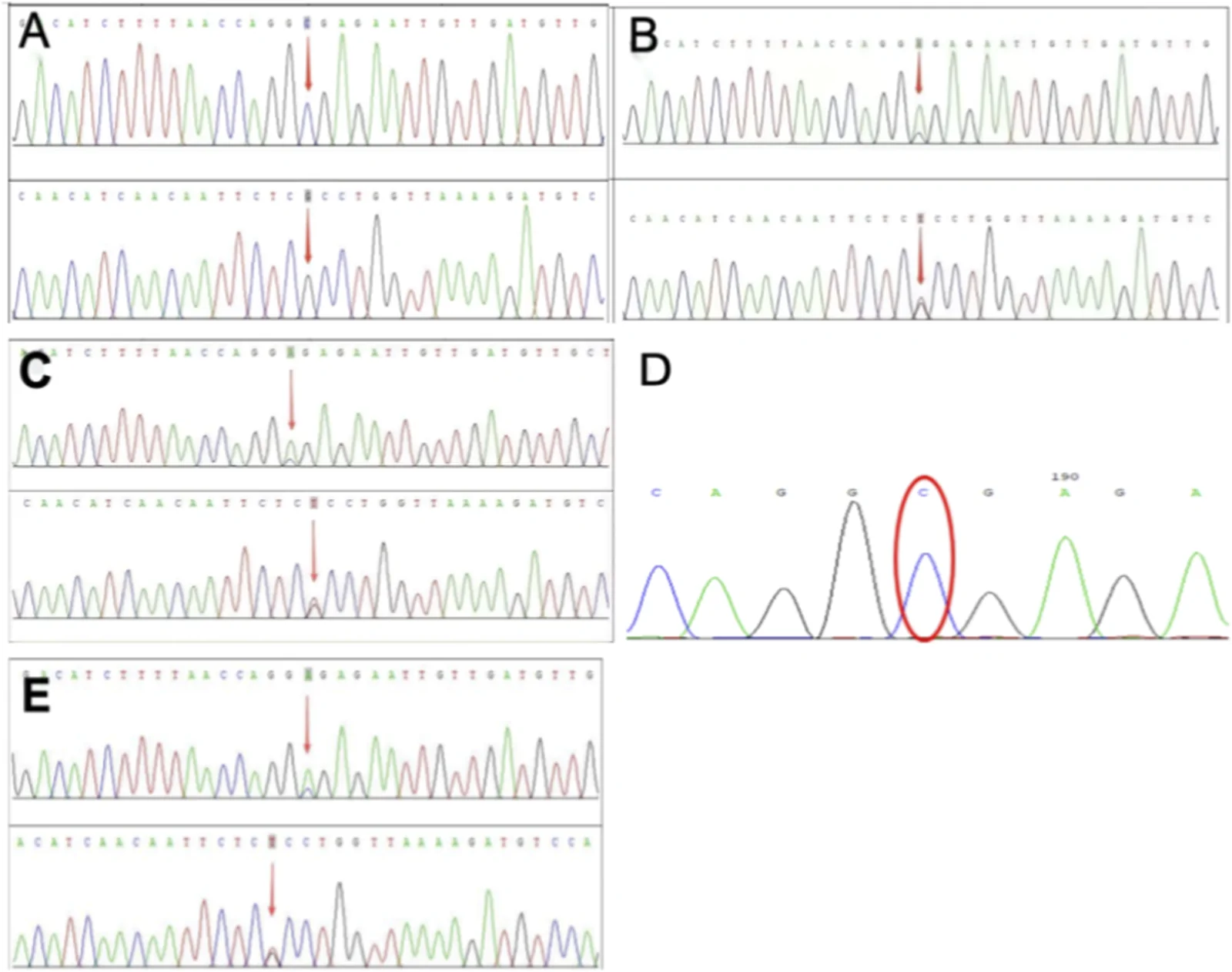
GLA gene sequencing in the proband and family members. Note: GLA Sanger sequencing in the proband **(A)**, daughters **(B,C)**, brother **(D)**, and niece **(E)**. The arrow indicates the variant position in the chromatogram.

Cardiac amyloidosis was considered unlikely based on the available findings. Transthoracic echocardiography demonstrated marked left ventricular wall thickening, raising suspicion for an inherited hypertrophic cardiomyopathy, including FD. Therefore, genetic testing was pursued. Whole-exome sequencing (Table 1) identified a previously unreported missense variant in the GLA gene, c.752A>C (p.Glu251Ala). This variant has not been reported in population databases or the published literature and is therefore considered novel. Its clinical significance remains uncertain, as its impact on α-Gal A activity and disease progression has not been functionally characterized. Sanger sequencing confirmed the presence of the c.752A>C variant in the proband. Following genetic counseling, cascade testing identified the same variant in multiple family members, which is consistent with co-segregation of the variant within the family (Figure 4).

**TABLE 1:** Whole-exome sequencing of the proband.

Gene	Chromosomal location	Variant (HGVS)	Zygosity (proband)	Inheritance	Associated disease (OMIM)	Variant origin	Classification (ACMG/AMP)	NGS reads (Ref/Alt)	VAF (%)
GLA	chrX:100653822	NM_000169.3:c.752A>C(p.Glu251Ala)	Hemizygous	X-linked	FD (OMIM: 301500); FD, cardiac variant (OMIM: 301500)	Unknown	VUS	207/33	13.8*

GLA, encodes α-galactosidase A; potentially associated variants in GLA, cause Fabry disease. Chr, chromosome; HGVS, human genome variation society; VUS, variant of uncertain significance; VAF, variant allele fraction (alternate reads/total reads).

* Although the variant is described as hemizygous, the observed VAF (13.8%) is lower than expected for an X-linked variant in a male; see Discussion for details.

### Biochemical findings

As shown in Table 2, the patient’s plasma lyso-Gb3 level was elevated, and α-Gal A activity was markedly reduced. The patient’s older daughter also had an elevated lyso-Gb3 level with decreased α-Gal A activity. Color Doppler echocardiography of the older daughter showed no significant myocardial thickening, and speckle-tracking echocardiography revealed no apparent abnormalities in global longitudinal strain (GLS). The patient’s younger daughter had an elevated lyso-Gb3 level, whereas her α-Gal A activity was within the normal range. The patient’s older brother, tested in another laboratory, likewise showed an elevated lyso-Gb3 level and decreased α-Gal A activity. The brother’s daughter also underwent enzyme testing, which demonstrated an elevated lyso-Gb3 level and reduced α-Gal A activity. Collectively, these biochemical findings, together with the genetic data, support the possibility that this variant is disease-associated in FD. Notably, this variant has not previously been reported as a potentially associated variant.

**TABLE 2:** α-Galactosidase A (α-Gal A) activity and globotriaosylsphingosine (Lyso-Gb3) levels in the proband and family members.

Individual	Relationship	Sex	Age (years)	α-Gal A activity (nmol/h/mg)	lyso-Gb3 (nmol/L)	GLA genotype (c.752A>C)
A	Proband	Male	54	7.03	79.05	+
B	Daughter	Female	26	31.77	11.68	+
C	Daughter	Female	20	49.38	7.12	+
D	Brother	Male	55	1.55*	23.78*	+
E	Niece	Female	22	27.92	16.37	+

Reference ranges: α-Gal A >34.76 nmol/h/mg; lyso-Gb3 <2.54 nmol/L.

* Results for individual D were obtained from a different laboratory using different units and reference ranges and are not directly comparable (reference range:α-Gal A, 2.20–17.65umol/L/h; lyso-Gb3 <1.11 ng/mL).“+” indicates presence of the c.752A>C variant.

### Pedigree analysis

**FIGURE 5:**
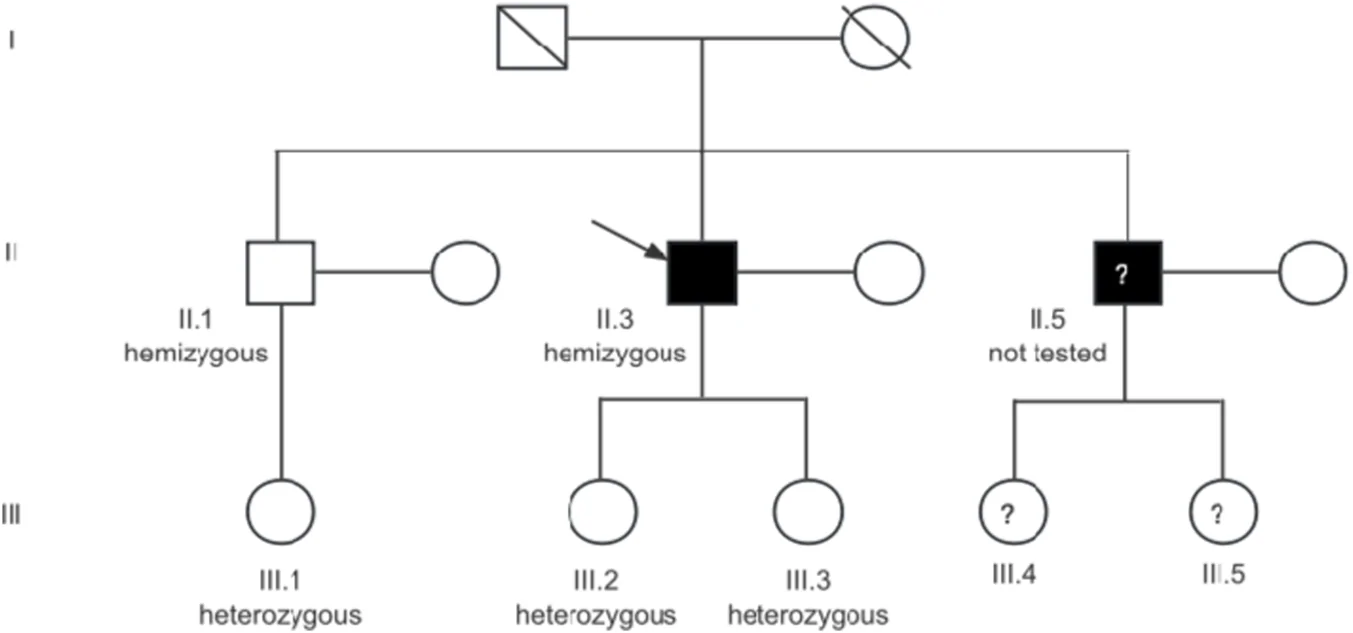
Pedigree of the family with the GLA c.752A>C (p.Glu251Ala) variant. Notes: Squares indicate males and circles indicate females. Filled symbols indicate individuals with cardiac involvement (e.g., LVH), while open symbols indicate those without clinical manifestations. Genotype is indicated below each individual (hemizygous or heterozygous). Symbols with a question mark indicate individuals who were not genetically tested. A diagonal slash indicates deceased individuals. The arrow indicates the proband. LVH, left ventricular hypertrophy.

A three-generation pedigree was constructed based on family investigation and genetic testing results (Figure 5). Squares denote males and circles denote females, and the arrow indicates the proband (II.3). Filled symbols represent individuals with cardiac involvement (e.g., LVH), while open symbols indicate those without clinical manifestations. Individuals who have not undergone genetic testing are indicated as having unknown genetic status. The proband (II.3) was found to carry a hemizygous GLA variant (c.752A>C, p.Glu251Ala) and presented with LVH. Another male sibling (II.1) was also confirmed to carry the same variant but clinical phenotype unknown. In contrast, a third male sibling (II.5) did not undergo genetic testing but demonstrated LVH on echocardiography. In the third generation, all tested daughters of variant-positive males were found to carry the same variant. Specifically, III.1 (daughter of II.1) and III.2 and III.3 (daughters of II.3) were heterozygous for the c.752A>C variant and showed no clinical manifestations at the time of evaluation. Two individuals in the third generation (III.4 and III.5), daughters of II.5, have not undergone genetic testing and therefore their variant status remains unknown. The parents in the first generation (I.1 and I.2) are deceased, and their genotypes are unavailable. However, the presence of the variant in multiple male siblings suggests that maternal transmission cannot be excluded. Overall, the observed segregation pattern is consistent with X-linked inheritance and demonstrates co-segregation of the GLA variant with the clinical phenotype within this family, providing supportive—though not definitive—evidence for its potential pathogenicity.

Clinical characteristics of genotype-positive family members are summarized in Table 3. The proband (II-3), a 54-year-old male, presented with chest pain and dyspnea and showed overt cardiac involvement, including LVH (maximum wall thickness of 18 mm) on echocardiography, accompanied by corresponding electrocardiographic abnormalities. Enzyme replacement therapy was subsequently initiated. In contrast, his brother (II-1), despite harboring the same GLA c.752A>C variant, had an undetermined clinical phenotype due to the lack of comprehensive clinical evaluation. Similarly, three female carriers (III-1, III-2, and III-3) were asymptomatic, with no evidence of cardiac or renal involvement at the time of assessment. Notably, another male family member (II-5), who had not undergone genetic testing, exhibited LVH on echocardiography, raising the possibility of unrecognized disease involvement. Overall, these findings highlight marked intrafamilial phenotypic heterogeneity associated with the GLA c.752A>C variant, characterized by severe cardiac involvement in the proband, absent or subclinical manifestations in other genotype-positive individuals, and a potential sex-related difference in disease expression.

**TABLE 3:** Clinical characteristics of genetically positive family members.

Individual	Sex	Age (years)	Genotype	Symptoms	Organ involvement	ECG findings	Echocardiography	Renal biomarkers	Treatment
Proband (II-3)	M	54	c.752A>C	Chest pain, dyspnea	Cardiac	LVH	LV wall thickness 18 mm	Normal	ERT
II-1	M	55	c.752A>C	—	—	Unknown	Unknown	Unknown	Unknown
III-1	F	22	c.752A>C	—	—	Normal	Normal	Normal	None
III-2	F	26	c.752A>C	—	—	Normal	Normal	Normal	None
III-3	F	20	c.752A>C	—	—	Normal	Normal	Normal	None

LVH: left ventricular hypertrophy; ERT: enzyme replacement therapy; eGFR: estimated glomerular filtration rate.

## Discussion

FD is an X-linked lysosomal storage disorder characterized by deficient α-Gal A activity, resulting in progressive glycosphingolipid accumulation and multisystem involvement. Cardiac involvement is a key determinant of disease burden and prognosis, most commonly presenting as LVH with associated structural and functional abnormalities (Ortiz et al., 2018; Zarate and Hopkin, 2008; Linhart and Elliott, 2007; Kampmann et al., 2008).

In this study, we describe a 53-year-old male presenting with LVH, markedly reduced α-Gal A activity, and elevated lyso-Gb3 levels. Genetic analysis identified a previously unreported GLA variant, c.752A>C (p.Glu251Ala). Family investigation suggested co-segregation of the variant with biochemical abnormalities and cardiac involvement, providing supportive—but still limited—evidence for a possible genotype–phenotype association. This observation may expand the mutational spectrum of the GLA gene and raises the possibility that this variant is associated with a cardiac-predominant phenotype. In addition, the combined use of biochemical, imaging, and familial data may help to improve the interpretation of variants of uncertain significance in FD, although the strength of such evidence remains inherently limited. Notably, the identification of the same GLA variant in multiple family members allows for a preliminary exploration of phenotypic variability. While cardiac involvement appears to be relatively common among carriers, differences in disease severity and biochemical profiles were observed. These findings are broadly consistent with the known clinical heterogeneity of FD and highlight the challenges of establishing clear genotype–phenotype correlations, particularly in the context of variants of uncertain significance.

Fabry disease–related cardiomyopathy should be differentiated from other causes of LVH, particularly sarcomeric hypertrophic cardiomyopathy (HCM), Danon disease, and PRKAG2 syndrome. Unlike HCM, which is primarily caused by mutations in sarcomeric protein genes and typically presents with myocyte disarray and asymmetric septal hypertrophy, FD is characterized by lysosomal glycosphingolipid accumulation, often accompanied by concentric hypertrophy and low native T1 values on cardiac magnetic resonance imaging. Danon disease, an X-linked disorder due to LAMP2 deficiency, shares features such as early-onset hypertrophy and conduction abnormalities but is usually associated with more severe skeletal myopathy and cognitive impairment. PRKAG2 syndrome, caused by mutations affecting AMP-activated protein kinase, can also present with ventricular hypertrophy and pre-excitation; however, glycogen accumulation rather than Gb3 deposition underlies its pathophysiology. Accurate differentiation among these entities is essential, as their management strategies and prognostic implications differ substantially (Nicholls, 2014; Hong et al., 2023; Arad et al., 2005; Nordin et al., 2019b).

In the present case, the absence of key distinguishing features—such as pre-excitation, skeletal myopathy, multisystem involvement, or a clear alternative genetic diagnosis—makes these conditions less likely. However, given the atypical imaging findings and the identification of a variant of uncertain significance (VUS), these alternative etiologies cannot be completely excluded.

Due to the clinical heterogeneity and nonspecific manifestations of FD, diagnosis can be challenging. Factors such as skewed X-chromosome inactivation further complicate interpretation, particularly in female patients, where α-Gal A activity may be variable. Therefore, molecular genetic testing of the GLA gene remains essential for diagnostic confirmation (Schiffman et al., 2017). To date, more than 1,000 GLA variants have been reported, encompassing missense, nonsense, frameshift, and splice-site variants. However, pathogenic or likely pathogenic variants are identified in only approximately 80% of clinically suspected cases, leaving a substantial proportion without definitive molecular confirmation. The IVS4+919G>A splice variant is relatively common among Chinese patients with late-onset FD (Hwu et al., 2009), while variants such as p.Asn215Ser, p.Met187Arg, and p.Ile239Met have been associated with predominantly cardiac phenotypes (Oder et al., 2017; San Román-Monserrat et al., 2014).

The c.752A>C (p.Glu251Ala) variant identified in this case results in substitution of a negatively charged glutamic acid with a nonpolar alanine residue, which may affect protein structure or stability. Although no functional studies have been performed, such a physicochemical change could potentially influence enzyme folding, catalytic activity, or intracellular trafficking. Nevertheless, these effects remain speculative in the absence of experimental validation.

According to ACMG/AMP criteria, this variant is classified as a VUS. It fulfills PM2 (absence or rarity in population databases), PP1 (co-segregation with disease phenotype), and PP4 (phenotype highly specific for FD, supported by reduced enzyme activity and elevated lyso-Gb3). However, stronger evidence is lacking, including functional validation (PS3), previously established pathogenic variants at the same residue (PS1/PM5), or robust computational/structural evidence. Therefore, its pathogenicity remains uncertain (Richards et al., 2015).

Importantly, an inconsistency was observed between the hemizygous status of the proband and the relatively low variant allele fraction (VAF) detected by next-generation sequencing (13.8%, 33/240 reads). For an X-linked variant in a male, a VAF close to 100% would typically be expected. Potential explanations include technical factors such as amplification bias or mapping artifacts, as well as the possibility of somatic mosaicism. Although the variant was confirmed by Sanger sequencing, supporting its presence, the atypical VAF introduces additional uncertainty regarding its biological origin and should be considered when interpreting its clinical significance. The absence of histopathological confirmation represents an additional source of diagnostic uncertainty, particularly in the context of atypical imaging findings and a VUS.

From a clinical perspective, early recognition of FD is critical, as timely initiation of ERT can improve outcomes. Recombinant α-Gal A has been available since 2001 and has been shown to stabilize or reduce left ventricular mass and improve functional capacity (Azevedo et al., 2020; Kampmann et al., 2015). Migalastat, a pharmacological chaperone, has also demonstrated efficacy in reducing left ventricular mass in selected patients (Germain et al., 2016). However, once myocardial fibrosis is established, it is generally considered irreversible, underscoring the importance of early diagnosis. Despite identification of this novel variant, VUS findings remain common in FD and complicate clinical decision-making. While family co-segregation analysis in this case provides supportive evidence, it is insufficient to establish causality. The patient has initiated agalsidase therapy and remains clinically stable; continued follow-up is warranted.

In summary, this case underscores the potential utility of a multimodal diagnostic approach integrating clinical, imaging, biochemical, and genetic data in the evaluation of suspected FD. The c.752A>C (p.Glu251Ala) variant remains classified as a variant of uncertain significance, and the available evidence suggests a possible association with the observed phenotype. However, the current data are insufficient to support a causal relationship. Further studies, including functional investigations and validation in larger cohorts, are warranted to clarify the clinical significance of this variant.

## Limitations

This case report has several limitations. First, it describes a single patient and his family, which limits the generalizability of the findings. Validation of the identified GLA variant in larger FD cohorts is needed to determine its prevalence and clinical relevance across different populations. Second, the variant identified in this study (p.Glu251Ala) remains classified as a variant of VUS, and no functional studies were performed. In particular, the effects of this variant on α-Gal A activity and lyso-Gb3 accumulation were not assessed. Therefore, the potential pathogenic mechanism remains unclear, and further investigations, including *in vitro* and/or *in vivo* functional studies, are required. Third, although the variant was detected in family members, comprehensive phenotypic characterization and long-term follow-up are lacking. Such data would be valuable for evaluating genotype–phenotype correlations, disease progression, and the response to ERT in this specific genetic context. Finally, a key limitation is the absence of histopathological confirmation. Endomyocardial biopsy, although considered the gold standard for diagnosing FD, was not performed due to its invasive nature and associated procedural risks. In this case, the diagnosis was based on clinical features, imaging findings, and genetic testing; however, the imaging pattern was atypical and the identified variant is a VUS. Therefore, the lack of tissue confirmation limits the ability to establish a definitive diagnosis and to confirm the pathogenicity of the variant. These limitations should be taken into account when interpreting the findings. Although non-invasive diagnostic approaches are increasingly used in clinical practice, histological confirmation remains particularly valuable in atypical presentations or genetically inconclusive cases.

## Conclusion

This study describes a novel GLA variant (c.752A>C; p.Glu251Ala) identified in multiple individuals from the same family presenting with LVH and clinical features suggestive of FD. The combination of biochemical abnormalities and familial co-segregation provides supportive—although not definitive—evidence for a potential genotype–phenotype association, thereby expanding the molecular and clinical spectrum of GLA variants. However, as the variant remains classified as a variant of VUS, its pathogenic role cannot be conclusively established. Further studies, including functional validation and investigation in larger cohorts, are required to clarify its molecular effects and clinical relevance.

## Data Availability

The original contributions presented in the study are included in the article/supplementary material, further inquiries can be directed to the corresponding author.
